# Supplementary material for: Memory shapes microbial populations
Source: PLoS Comput Biol. 2021 Oct 1;17(10):e1009431. doi: 10.1371/journal.pcbi.1009431 (PMC8513827; doi:10.1371/journal.pcbi.1009431)
Supplement: S1 Text — (PDF) [file pcbi.1009431.s001.pdf]

Supplementary Information: Memory shapes microbial populations

Chaitanya S. Gokhale[1*], Stefano Giaimo[2], Philippe Remigi[3]

1 Research Group for Theoretical Models of Eco-evolutionary Dynamics, Department of Evolutionary Theory, Max-Planck Institute for Evolutionary Biology, Plön, Germany
2 Department of Evolutionary Theory, Max-Planck Institute for Evolutionary Biology, Plön, Germany
3 LIPME, Universite de Toulouse, INRAE, CNRS, Castanet-Tolosan, France

*gokhale@evolbio.mpg.de

Matrix model and solution.

The system in Eq. 2 of the main text can be written in matrix form as

$$\frac{\mathrm{d}\boldsymbol{x}}{\mathrm{d}t} = \boldsymbol{A}\boldsymbol{x}(t), \qquad \boldsymbol{x}(0) = \boldsymbol{x}_0 \tag{1}$$

where

$$\boldsymbol{A} = \begin{pmatrix} b_0 - d_0 - \mu & & & & \epsilon \\ \mu & b_n - d_n - \epsilon & & & \\ & \epsilon & \ddots & & \\ & & \ddots & \ddots & \\ & & & \epsilon & b_1 - d_1 - \epsilon \end{pmatrix} \tag{2}$$

and $\boldsymbol{x}(t) = (x_0, x_n, \ldots, x_1)^\top$, here the superscript T indicates vector transposition. The solution to Eq. 1 is $\boldsymbol{x}(t) = e^{t\boldsymbol{A}}\boldsymbol{x}_0$. The matrix $\boldsymbol{A}$ is essentially non-negative (i.e. off-diagonal entries are non-negative) and irreducible (i.e. any compartment can be reached from any other compartment). This ensures that $\boldsymbol{A}$ has a real dominant eigenvalue λ such that all other eigenvalues have smaller real part. As a consequence, asymptotically ($t \to \infty$) the cell population grows exponentially with rate λ and the distribution of cells in the compartments approaches the right eigenvector $\boldsymbol{u}$ corresponding to λ, when this eigenvector is scaled so that its components add up to unity.

Symmetric growth dynamics.

Under complete symmetry in the growth dynamics for all compartments, i.e. $b_i = b$ and $d_i = d$, we have that $\lambda = b - d$, i.e. growth equals birth rate minus death rate. Solving then the eigenvector equation $\boldsymbol{A}\boldsymbol{u} = \lambda\boldsymbol{u}$ for the symmetric case, the equilibrium fraction of cells in any 'on' compartment ($i = n, n-1, \ldots, 1$) is equal to $\mu/(\epsilon + n\mu)$. Thus, the equilibrium fraction of cells in the 'off' state, i.e. in the zeroth compartment, is equal to $\epsilon/(\epsilon + n\mu)$. This expression shows that a change in the number of 'on' compartments and a change in the ratio of the switching rate to the leaching rate have the same effect on this equilibrium: if n or the μ-to-ϵ ratio increase (decrease), the equilibrium fraction of cells in the 'on' state increases (decreases). However, the equivalence breaks down

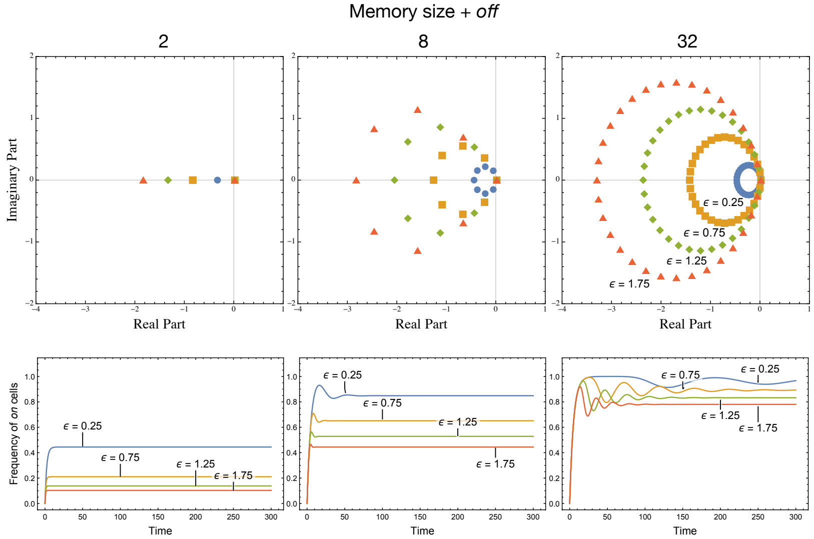

Fig A. Eigenvalue spectra and population dynamics. ($b_i = 1.0$ and $d_i = 0.98$ for all $i = 0, n, n-1, \ldots, 1$) for different number of compartments (Memory $n = 1, 7, 31$ + 1) and different values of the leaching rate ($\epsilon = \{0.25, 0.75, 1.25, 1.75\}$). The switching rate ($\mu = 0.1$) is kept constant. This behaviour is related to the spectra of the matrix models (top) that govern dynamics (bottom), especially the overshooting.

when one looks at transient dynamics (Fig. A). To understand why, let $\lambda_0, \lambda_1, ..., \lambda_n$ be the eigenvalues of $\boldsymbol{A}$ in Eq. 2 in order of decreasing real part and $\boldsymbol{u}_0, u_1, ..., \boldsymbol{u}_n$ be the corresponding eigenvectors, which are assumed linearly independent. Then, the solution to Eq. 1 can be written as

$$\boldsymbol{x}(t) = \sum_{j=0}^{n} a_j e^{t\lambda_j} \boldsymbol{u}_j \tag{3}$$

where a_j are fixed scalars corresponding to the coordinates of $\boldsymbol{x}_0$ in the basis given by the eigenvectors of $\boldsymbol{A}$. This solution highlights how subdominant eigenvalues may influence dynamics for small t, i.e. before the asymptotic phase when these get dominated by λ_0. The evolution of $\boldsymbol{x}(t)$ relative to the influence of the dominant eigenvalue over time is given by $\boldsymbol{x}(t)/e^{t\lambda_0} = \sum_{j=0}^{n} a_j (e^{t\lambda_j}/e^{t\lambda_0})\boldsymbol{u}_j$. Since λ_0 dominates the other eigenvalues, we have that $|e^{\lambda_j}/e^{\lambda_0}| < 1$ for $j > 1$ and we get that $\boldsymbol{x}(t)/e^{t\lambda_0} = a_1 \boldsymbol{u}_j$ as $t \to \infty$. But for small t, the quantities $a_j(e^{t\lambda_j}/e^{t\lambda_0})\boldsymbol{u}_j$ may be not negligible. Typically, the closer subdominant eigenvalues are in their real part to the dominant eigenvalue, the larger and more protracted the transients. Complex eigenvalues also introduce oscillations. In our case, as Fig. A shows, the more 'on' compartments are present, the more complex eigenvalues that are closer in real part to the dominant are found in the spectrum of $\boldsymbol{A}$. Dynamically, this produces greater overshooting and slower convergence. This is not observed when the μ-to-ϵ ratio increases yet a single compartment is present. For $n = 1$, the only subdominant eigenvalue is $\lambda - \mu - \epsilon$, which is real and gets closer to the dominant eigenvalue λ as the μ-to-ϵ ratio increases. But while this increase leads to an increase in the equilibrium fraction of 'on' cells, see formula above, no overshooting ensues, as Fig. **??** in the main text shows.

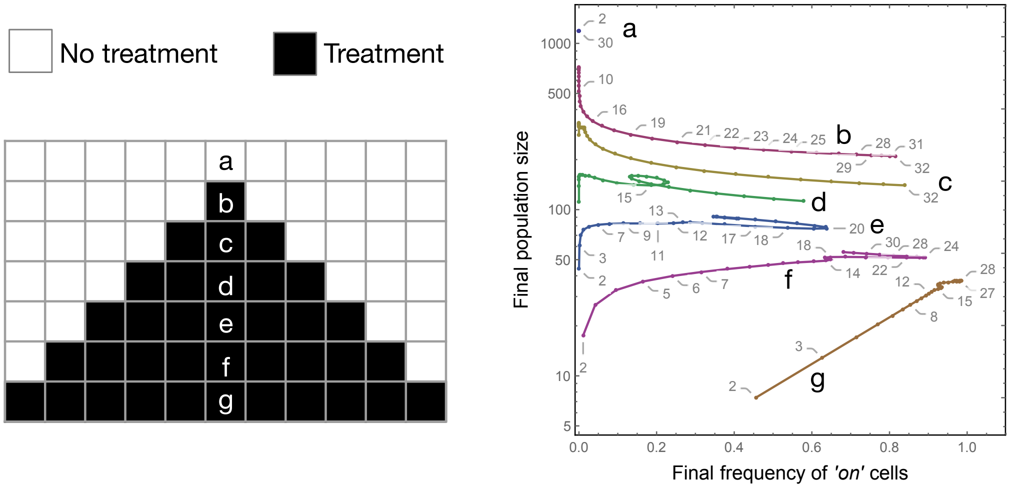

Fig B. Final frequencies of the ‘on’ cells and the final population sizes of lineages with different memory lengths. The results of the population and evolutionary (frequency change over time) dynamics of the cell lineages with different memory sizes ($n = (1, \ldots, 31)$)+1 are plotted after different sequences of environments each comprising of 11 seasons. There are two kinds of seasons - treatment (filled) and no treatment (empty). Each season lasts for 15 numerical time-steps The sequences from (a) to (g) increase the length of antibiotic treatment where the ‘off’ cells die with rate $b_{off} - d_{off} = 1 - 1.02 = -0.02$. The ‘on’ cells do not grow. In the absence of antibiotics there is no switching from ‘off’ to ‘on’ i.e. $\mu_{\text{no treatment}} = 0$ whereas treatment triggers the switch $\mu_{\text{treatment}} = 0.2$.

Environmental Variation.

Populations of cells can respond to stressful environmental conditions in a variety of ways. We focus on sequences of environments composed of multiple seasons as a source of environmental stress. Each season is either conducive or harmful to the population. As a response, a lineage of cells is bestowed with different growth rates for the ‘off’ and ‘on’ compartments. The ‘off’ cells thus correspond to the growing, typically the observable quantity of a lineage. The ‘on’ cells do not grow but flow through the compartments. We look at ‘triggered’ persistence [**?**], i.e. the cells switch from ‘off’ to ‘on’ only when the season is harmful.

Sequence diversity. As an example of controlled fluctuating environments, antibiotic treatment schedules are apt examples. Treatment regimes take various forms [1, 2]. A good understanding of the effect of sequence and even sequence memory of cells can help design effective treatment regimes aimed at minimising collateral resistance to multiple drugs.

In the main text we focused on a large number of treatment sequences (in total 84). The sequences ranged from no treatment to all treatment where each season lasted one time unit. The growth rates of lineages of cells with different memories was calculated as $g_m(i) = \log(N_{tmax}/N_0)/t_{max}$ for each memory size i. We compare this Malthusian growth rate to that of a lineage which gives the same eventual ‘on’ frequency but for $n = 1$ i.e. a memoryless process (which will have a different leaching rate ϵ). Thus $r = g_m(i) - g_{mless}(n = 1, \epsilon)/t_{max}$ is the difference in the Malthusian growth rates of the two lineages if under direct competition (`http://myxo.css.msu.edu/ecoli/srvsrf.html`).

Using our minimal setup we can explore the effects of relaxing treatment for different lengths of time. However it would be useful to reduce the number of sequence

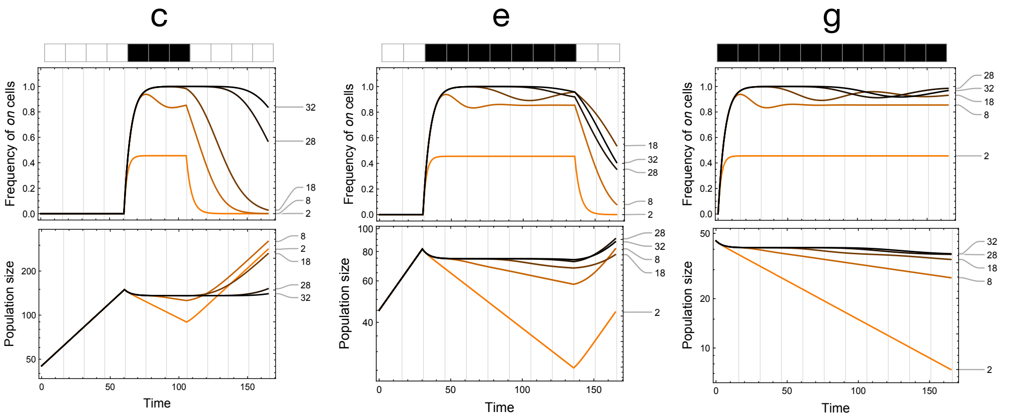

Fig C. Lineage dynamics with different memories under select treatment regimes. To better understand the non-linear behaviour seen in the Fig. B we choose three different treatment regimes (c), (e) and (g). The plot denote the temporal dynamics of (top) the fraction of 'on' cells and the (bottom) population size for lineages with different memory sizes ($n + \text{off} = 2, 8, 18, 28, 32$). Under no treatment (empty) the 'off' cells grow with rate $b_{off} - d_{off} = 1 - 0.98 = 0.02$ and there is no switching from 'off' to 'on' ($\mu_{\text{no treatment}} = 0$). Under antibiotic treatment the 'off' cells die with rate $b_{off} - d_{off} = 1 - 1.02 = -0.02$ and switching occurs with rate $\mu_{\text{treatment}} = 0.2$. The 'on' cells do not grow. For treatment sequence (c) we have a short treatment length. The memoryless lineage ($n + 1 = 2$) reaches equilibrium in 'on' cells immediately. Lineage with 8 compartments shows a characteristic over and undershoot in the short treatment time whereas the others have longer amplitudes and the treatment is not long enough to see them. When the treatment is finished the shorter memory lineages have a shorter time lag in resuming growth. For treatment sequence (e) the transients of the longer memories show the over and undershoots affecting the final on proportions. Also since there in not much time left after the treatment ends, the shorter memory lineages do not have enough time to increase in population size. Under sustained treatment in (g) the 'on' cell equilibrium is being approached but the sequence length is not enough for all lineages to achieve it. Only the memoryless and the lineage with 8 compartments reach equilibrium while the others still show oscillations. Under sustained treatment the lineages decline in population size but a larger memory buffers the time of decline. The initial population consist of all 45 all 'off' cells. Each season (treated or untreated) lasts for 15 numerical time-steps. The leaching rate is set to $\epsilon = 0.25$.

to perform a thorough analysis. In the main text the fluctuating sequences last for 165 time-steps leading to 84 treatment regimes. We let each season last for 15 time-steps instead of 1, which yields us only 7 different sequences of 11 seasons each. The analysis of these sequences is shown in Fig. B and Fig. C.

Condition dependent switching. In the main text we have assumed 'triggered persistence' i.e. when not under treatment the switch from 'off' to 'on' does not work. Relaxing this assumption, in Fig. D, we show the result of the dynamics on the spectrum of constitutive switching where ($\mu_{\text{treatment}} = \mu_{\text{no treatment}} = 0.2$) to strictly antibiotic triggered switching ($\mu_{\text{treatment}} = 0.2, \mu_{\text{no treatment}} = 0.0$). If a lineage switches to the 'on' cells constitutively then the eventual population size is much smaller as the lineage loses cells to the non-growing state and the growth can only proceed after they have exited the 'on' cells after leaching through all the memory states. The exact shape of the plotted clines depend further on the specific sequence chosen to be explored.

Long term growth dynamics for cyclic environments. Using the analysis

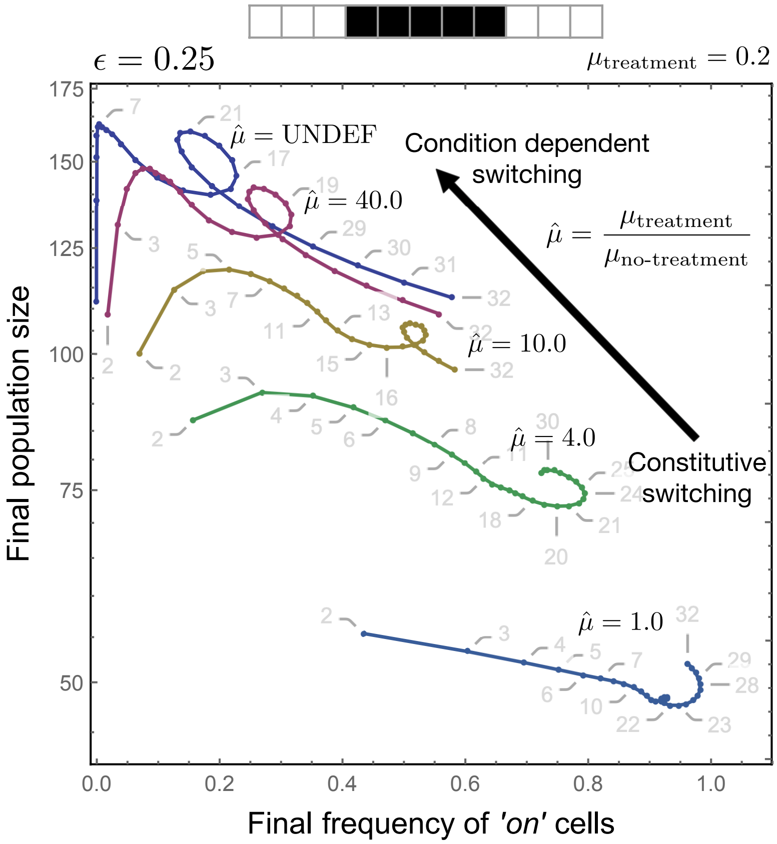

Fig D. Constitutive switching to "triggered" persistence. For the antibiotic tolerance we assumed that the 'on' cells are produced only when under treatment ($\mu_{treatment} = 0.2$ and $\mu_{no-treatment} = 0.0$). Here we explore the effect of relaxing this assumption. We define $\hat{\mu}$ as the ratio between $\mu_{treatment}$ and $\mu_{no-treatment}$. For constitutive switching i.e. switching irrespective of the environment, the 'on' cells can saturate for longer memories, however since the 'on' cells do not grow, the population sizes are low. As we move towards condition dependent switching, the population is rescued with the final population size showing a prominent non-linear dependence on the memory size n (grayed out numbers showing $n+1$). Also since 'on' cells are produced only under treatment and they do not grow, the final frequency of 'on' cells is lower than when under constitutive switching.

in [3], we also consider long term dynamics when the population goes through the same seasonal sequence a repeated number of times. We form two matrices $\boldsymbol{A}_\mathrm{R}$ and $\boldsymbol{A}_\mathrm{T}$ with the same structure as in Eq. 2. Matrix $\boldsymbol{A}_\mathrm{R}$ is parametrized to reflect the growth and phenotypic dynamics of the population under a relaxed season, while matrix $\boldsymbol{A}_\mathrm{T}$ is parametrized to reflect the growth and phenotypic dynamics of the population under a treatment season. Given an initial population composition $\boldsymbol{x}(0)$, the final population composition $\boldsymbol{x}(n\tau)$ after a sequence comprising n seasons each lasting τ units of time is obtained as

$$\boldsymbol{x}(n\tau) = \underbrace{e^{\tau \boldsymbol{A}_{i_n}} \dots e^{\tau \boldsymbol{A}_{i_1}}}_{=\boldsymbol{B}} \boldsymbol{x}(0) \tag{4}$$

where $i_j \in \{\mathrm{R}, \mathrm{T}\}$, $j = 1, \dots, n$. When the population cycles m times through the same sequence, the population composition at the end of the m-th cycle is $\boldsymbol{x}(n\tau m) = \boldsymbol{B}^m \boldsymbol{x}(0)$. As m gets large, the population size grows by a factor equal to the dominant eigenvalue of $\boldsymbol{B}$ after having gone through a sequence. Using this approach, it can be shown (Fig. E) that short term population growth for different memory sizes as explored in Fig. 3 of the main text displays a strikingly qualitatively similar behavior as long term growth.

However, $\boldsymbol{B}$, as a product of matrices, has eigenvalues that are invariant to how matrices are arranged in this product. Therefore, this approach does not extend to our analysis in the next section where the effects of season permutations are explored.

Lag time distributions.

Upon sufficiently long exposure to sustained treatment, the population reaches a stable compartmental distribution given by the right dominant eigenvector $\boldsymbol{w}_\mathrm{T} = (w_0, \dots, w_n)^\top$ of the $\boldsymbol{A}_\mathrm{T}$ matrix scaled so that the eigenvector components add up to 1. This enables us to compute the lag time distribution when cells in 'on' state have no growth dynamics, i.e. $b_i - d_i = 0 - 0$ for $i = 0, \dots, n$, as assumed above. A randomly sampled cell from the exposed population is in compartment i with probability w_0. When the cell is in the zeroth compartment, i.e. 'off' state, its lag time is 0, as it is already dividing. When the cell is in any 'on' compartment, i.e. $i = 1, \dots, n$, its lag time is the time it takes for the cell to reach the 'off' state, i.e. the zeroth compartment, where division occurs. This time τ is gamma distributed with shape parameter i and rate parameter ϵ so that, a cell from compartment i has lag time τ with probability $\mathrm{Gamma}(i, \epsilon, \tau)$. The probability density $\mathrm{P}(\tau)$ that a sampled cell from the treated populations has lag time τ is then given by,

$$\mathrm{P}(\tau) = \sum_{i=1}^{n} w_i \mathrm{Gamma}(i, \epsilon, \tau). \tag{5}$$

which does not describe a gamma distribution.

Permutations. Another way of designing treatment regimes is to permute a given number of treatment seasons. As a template we look at the permutations of 4 treatment seasons and 4 relaxed seasons for a total of 70 possible sequences. We check the final size of a lineage once it has experienced all 8 seasons. We do so for cell lineages with different memory lengths. Plotting the final population size against the final 'on' cells frequency we see that an increase in the number of compartments typically results in an increase in the final 'on' frequency (but not always, see the loops), Fig. F. Also the population size peaks typically at intermediate memory length. An intermediate memory size can then be advantageous when selection operates on the surviving population at the end of the treatment sequence.

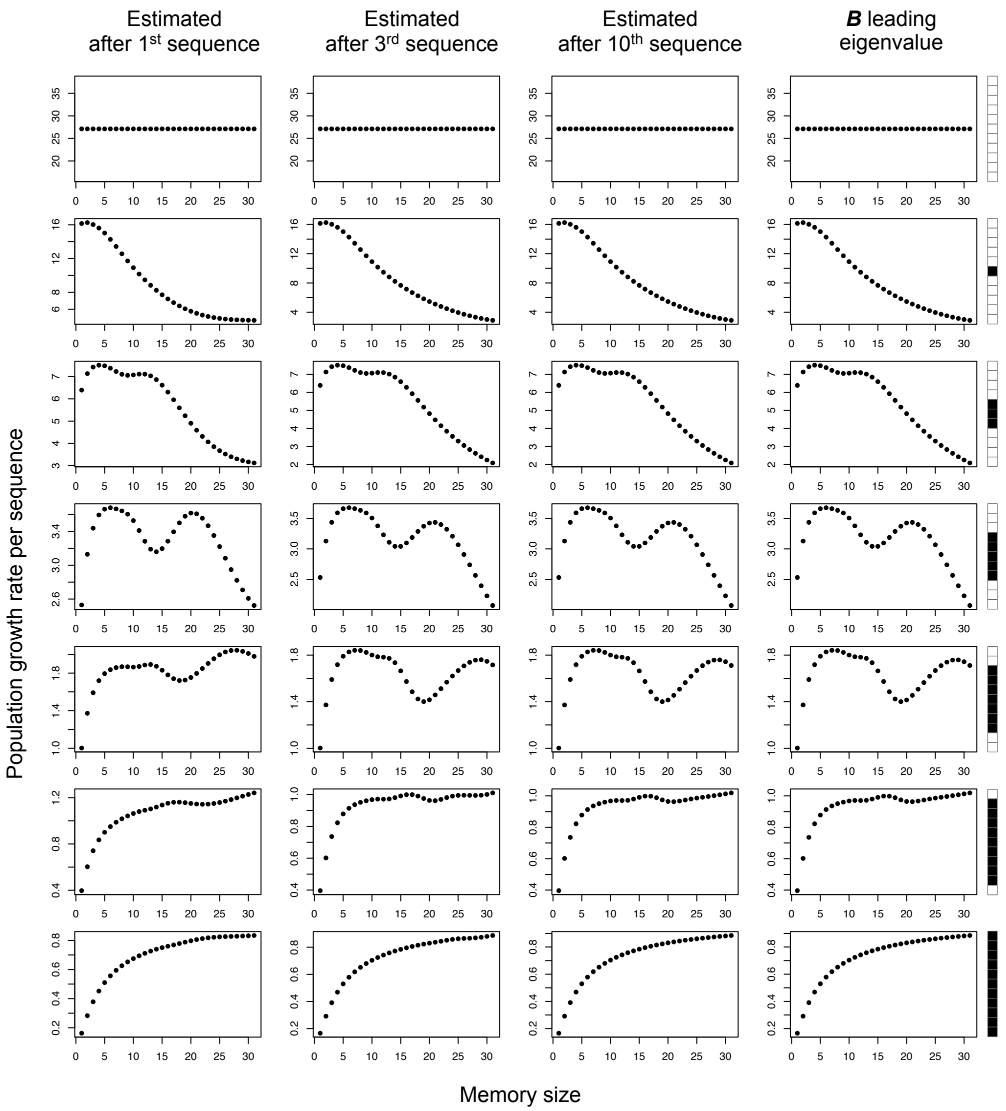

Fig E. Long term growth dynamics for cyclic environments Using the same parameters as in Fig. B, growth rate (y-axis) is estimated as the factor by which population has grown after having gone through one sequence of seasons. Cycling through the same sequence, the estimation is done after the first cycle, the third and the tenth. Resulting growth rates are compared with those expected in the long run as given by the leading eigenvalue of the matrix $\boldsymbol{B}$ that captures population dynamics over a sequence.

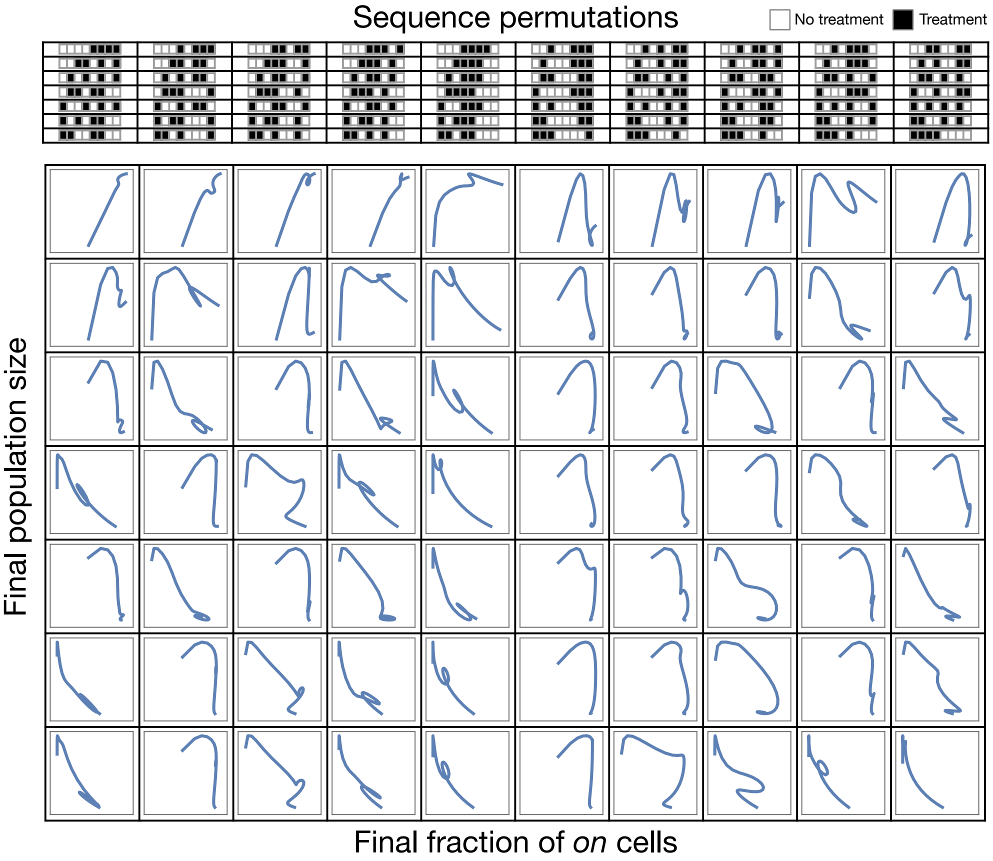

Fig F. Lineage outcomes for sequence permutations. For the 70 different permutations of the sequences consisting of 4 treatment seasons and 4 no treatment. Thus the total duration of the experiments is fixed while the continuous duration of the treatment can differ. Each of the the line-plots corresponds to each of the sequence permutations. The line-plots depict the final frequency of 'on' cells (x-axis) and the final population size (y-axis) as in Fig. C for lineages with different memory lengths ($n = (1, \ldots, 31)$). Under condition dependent switching ($\mu_{\text{treatment}} = 0.2$ else 0), parameters are $b_{off} - d_{off} = 1 - 0.98 = 0.02$ under no treatment and $b_{off} - d_{off} = 1 - 1.02 = -0.02$ under treatment. Each season (treated or untreated) lasts for 15 numerical time-steps. Leaching is constant at rate $\epsilon = 0.25$. Overall, we see that numerous sequences peak in final population for intermediate memory sizes and non-linearity is a general observable.

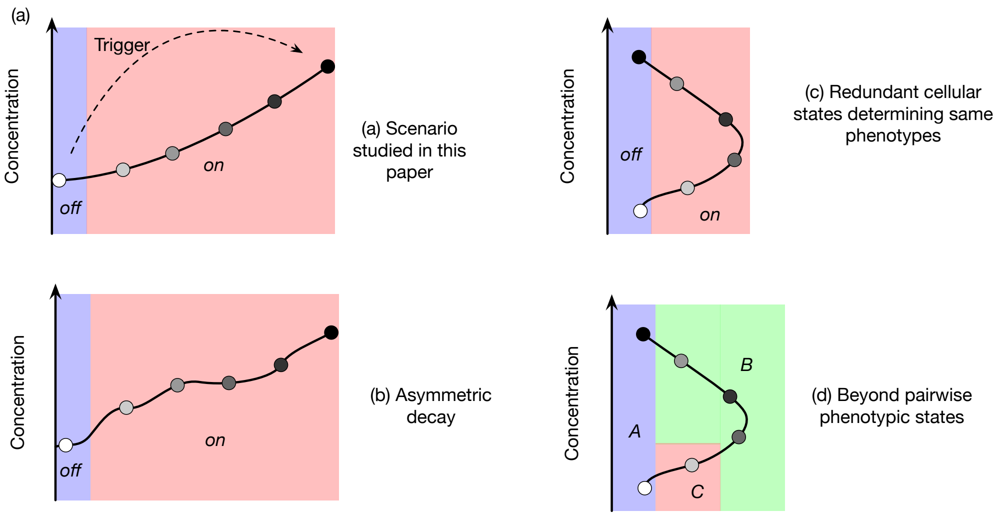

Fig G. Some complexities in phenotypic determinism An intracellular product, the presence and concentration of which depend on contingent ecological conditions, is a determinant of phenotypic state. (a) In our study we have assumed the simplest case where there are only two phenotypes, one of them characterised by multiple states. The decay of the determinant is smooth between the observed states and hence leads to tractable analytics. (b) It can be possible that the decay between the different tracked states is not smooth and this will affect the eventual distribution of phenotypes and the time spent in the two states. This situation can easily be captured in our framework by setting state dependent leaching rates, i.e. ϵ_i, in Eq. 2. (c) Even if the decay is smooth it is quite possible that the determinism incorporates redundancy. In this example, both low and high concentration leads to the same phenotype. To include this scenario in our framework, we envisage the addition of an additional state $n + 1$ that also leads to the 'off' phenotype and from which cells can switch to the previous state n acquiring the 'on' phenotype. (d) While two phenotypes are easier to handle both experimentally and theoretically, this is probably the result of studying two environments. Multiple phenotypes are a reality and for a complete understanding of phenotypic heterogeneity, multiple phenotypes need to be incorporated, as binary cases are often special cases.

Phenotypic determinism.

Phenotypes can be determined jointly by the internal states of a cell (e.g. intracellular protein concentrations) and external effects (available metabolites). Furthermore, the number of possible internal determinants can be numerous. Irrespective of the the possible complexity of phenotypic determinism, in our study we have assumed a system which is best depicted by Fig. G (a). A trigger (ecological- biotic or abiotic) or a stochastic process is assumed to increase the concentration of a certain phenotypic determinant that can be tracked. We have assumed only two phenotypic states - 'on' and 'off' . Also the decay landscape is gradual. As shown in Fig. G (a) it is possible that the decay process of the determinant is not smooth and (b) can lead to massive variation in the time spent in the two states. Fig. G (c) highlights the case when the concentration can determine the same two phenotypes in a redundant fashion. Both very low and very high concentrations generate the same phenotype 'off' whereas the intermediate is the 'on' state. Indeed an exact distinction between phenotypes is limited by the tests possible to differentiate between the phenotypes and their precision. Multiple phenotypes as shown in Fig. G (d) when considered will disrupt our classical analysis but will need to be included as molecular tools get more and more precise.

References

1. Fridman O, Goldberg A, Ronin I, Shoresh N, Balaban NQ. Optimization of lag time underlies antibiotic tolerance in evolved bacterial populations. Nature. 2014;513(7518):418–421.

2. Roemhild R, Gokhale CS, Dirksen P, Blake C, Rosenstiel P, Traulsen A, et al. Cellular hysteresis as a novel principle to maximize the efficacy of antibiotic therapy. Proceedings of the National Academy of Sciences. 2018;.

3. Kussell EE, Kishony RR, Balaban NQN, Leibler SS. Bacterial persistence: a model of survival in changing environments. Genetics. 2005;169(4):1807–1814.
