# Supplementary material for: Memory shapes microbial populations
Source: PLoS Comput Biol. 2021 Oct 1;17(10):e1009431. doi: 10.1371/journal.pcbi.1009431 (PMC8513827; doi:10.1371/journal.pcbi.1009431)

Memory shapes microbial populations
Reply to reviewers

Thanks for your submission. Both reviewers saw valuable work in this manuscript. However, both also raise important concerns. I am inviting the authors to submit a major revision of the manuscript, taking into account each of the reviewer comments. Particularly important I think are the comparison and contrasts of this model with existing internal compartment models; the relevance or justification of terms like evolution and memory; and the explanatory value of large vs small numbers of compartments.

We thank the editor and the reviewers for giving us the chance to respond to the queries and comments. We have specifically addressed the issues highlighted both in the reply itself as well as through edits in the manuscript, in the diff.pdf file in detail. We thank the editor and the reviewers for the thoughtful questions which we believe make the manuscript a better version. We hope you will agree.

Reviewer's Responses to Questions

Comments to the Authors:
Please note here if the review is uploaded as an attachment.

Reviewer #1: In the present manuscript the authors analyze a model of persister cells (bacteria) which implements a mechanism that differs from the standard on-off switching. In particular, they assume that rather than having the usual two states "on" and "off", the persistent state consists in a sequence of multiple intermediate "sub-states" or compartments (at some constant leaching rate). This means that the cell needs to go through a succession of stochastic steps before being able to turn back to the awaked state. Not surprisingly, this combination of individual processes leads to a non-exponential distribution of lag times, i.e. a Gamma distribution whose shape is controlled by the number of compartments (while for the case of just one compartment, the standard exponential distribution is recovered).

Then the authors move on to analyze a situation in which a community of such cells -- either with many compartments, i.e. with "memory", or with just one of them (standard on-off scenario)-- is exposed to antibiotics during some variable time interval.
First of all, it is found that effects such as overshoots emerge only in the presence of memory (stremming from complex eigenvalues in the eigenvalues controlling the

convergence to the equilibrium state. Then, the authors go on to show that there is a non-linear relation between memory length and absolute fitness; as could have been naively anticipated longer memories have a larger fit under longer antibiotic-exposure times and, conversely, for short antibiotic durations the tradeoff between survival and growth favors shorter memories. In particular, in this last case a strategy relying on a single compartment (with adapted leaching rate) can outperform memory, but, more generally, for a range of conditions memory outperforms classical memoryless switchning. They also elucidate a number of other properties of the model under different types of environments.

Overall the paper is clear, convincing and well written (well,...
english should be corrected, e.g. quite few "s" of verbs in third
person are missing). Thus, in principle, I would be tempted to recommend it for publication in PCB.

We thank the reviewer for the overall positive assessment of our work. Below we have taken your concerns into account and improved on the manuscript.

However, a number of important issues should be improved and clarified.

1) The authors do not cite the recent paper, published in PCB: "When to wake up? The optimal waking-up strategies for starvation-induced persistence", by Himeoka and Mitarai, that introduces a multistep process where "the cells sequentially go through multiple (M) dormant states" almost identical to the one proposed in the present manuscript. This work should be acknowledged and discussed.

Thanks for bringing to notice this very recent paper, that is indeed relevant to our study. Please note that our failure to include it in our original submission was due to how close it was published to our submission date. In our resubmission, we cite this work of Himeoka and Mitarai and we discuss the differences between their approach and ours. In particular, we note that Himeoka and Mitarai’s 2021 is an optimality study. They aim at computing the optimal lag time (time before resuming growth after having undergone a dormant state of cell cycle arrest that avoids the lethal drug effects) for bacterial populations that are exposed to antibiotic treatments. Hence, they aim at maximizing a certain objective function that may be interpreted as fitness. This function is a long-run growth rate and, as such, it describes an asymptotic state. In a small subsection of the

main text and their SI, Himeoka and Mitarai (2021) perform this fitness optimization task by including the possibility that cells may undergo multiple dormant states, instead of one state (their most central case). This subsection indeed shares similarities with our model, where cells go through several dormant (ON, in the parlance of our model) states. However, the overall approach differs between our work and theirs. The main reason being that we mostly focus on qualitative features of transient population dynamics (e.g., the presence of oscillations) and not, as we also emphasize in line 97 of the original submission on asymptotic properties, like the quantification and maximization of long term population growth, which is the main target of Himeoka and Mitarai's analysis (2021). Even where we evaluate population growth, we do so, by and large, over a single round of population transition through a deterministically varying environment. Therefore, this transient growth is not immediately informative about long term trends (but see our comparison with eigenvalue analysis of projection matrices). Although we cannot envisage a formal parallel, we find it encouraging that Himeoka and Mitarai (2021) observe a lack of monotonicity of long-run fitness in the multiple dormant state scenario as a function of different parameters (see their Figures A and B in the SI), as we do for transient/one-round fitness.

2) When a cell reproduces the offspring are assumed to be in the some state as the mother. Is this a reasonable biological assumption? For instance, if the leaching rate is related to the concentration of some protein, I would be tempted to believe that the numbers of such a protein are divided somehow among the two offspring, rather than being perfectly duplicated; i.e there should be a reduction in the protein numbers. Putting this question in more general terms: the cells are transmitting their state to their progeny; is this supposed to reflect some kind of epigenetic transmission of information? In any case, this choice should be better motivated/argued and put into context.

Interestingly in our very first drafts of the manuscript, we precisely had the same idea. However, after discussing with colleagues, experimentalists as well as theorists, we decided to decouple the phenotypic movement of cells from the growth dynamics which we realised could then confound the leaching rate. If we couple the two together, we get a slightly different model. It shows the same over(under) shoot properties qualitatively but we lose the causality of the movement of cells between compartments (growth and differentiation both contribute). This would be a good model for systems that show this coupling for e.g. Lord, N. D. *et al.* Stochastic antagonism between two proteins governs a bacterial cell fate switch. *Science* **366**, 116–120 (2019)). However, this approach is an

unwieldy tool for studying memory effects and prevents us from studying precisely those systems we target where no growth is expected (persistence mechanism) but only changes in phenotype are observed.
With such persistence based systems in mind, in our model, the cell does not divide but the protein concentration reduces by degradation or leakage. So we are talking about the same cell as it transitions through different "internal states".

For completeness, we have now attached our previous model where growth and leaching are connected as a supplementary file (and have added it to the Github repository). We followed the model inspired by the one described in *Werner, B., Dingli, D., Lenaerts, T., Pacheco, J. M. & Traulsen, A. Dynamics of Mutant Cells in Hierarchical Organized Tissues. Plos Comput Biol* ***7****, e1002290 (2011)* also published in PCB.
The model included in the main text as well as the one attached with this reply, in fact, point towards our discussion (and the Appendix) about complex phenotypic landscapes where there can be multiple ways to switch between on and off. The protein (or environment) dictating the phenotype could be a complex landscape as discussed in the Appendix.

However, given the number of extensions possible, if the reviewer and/or the editor feel it valuable then we would be happy to include this extension as a supplementary file.

3) The authors refer to the "evolutionary" part of the model as well as to the "eco-evolutionary" details. But, in the absence of mutation or variation in the inheritance process, I do not see any evolutionary process here: there are just phenotypic changes and "internal state" transitions. Maybe "collective adaptation" of the community to the imposed external conditions would be a less confounding name.

We agree with the reviewer here that we do competition trials but do not evolve the parameters. Our aim was to highlight the landscape consisting of the memory states n on which, evolution could then proceed as lineages compete. However, since going through that process is currently beyond the scope of this paper we reserve this topic only to the discussion and edit the figure titles accordingly. Thanks for the suggestion.

4) Similarly, the term "memory" sounds nice, but at the end of the day
what the model implements is a Markovian process. Moreover, "memory"
evoques a learning process where some number of compartments is selected after some environmental history. But here there is no such a learning process. In my opinion, it is not

well justified to call this effects a "memory"; even if I might be wrong, I think that this issue should be discussed more carefully in the manuscript.

Indeed, memory is a general term that may refer to different processes, including as used in learning dynamics. In this work, we use the formal description of microbial memory proposed by Norman et al. (Nature 2013; Ann Rev Microbiol 2015).
In the literature related to bacterial phenotypic heterogeneity, the term ‘memory’ has often been employed to refer to the phenomenon of hysteresis. Consider a gene whose expression can be induced by a chemical signal and which is involved in a positive feedback loop. Such a network can exhibit phenotypic heterogeneity (co-existence of ON and OFF cells, due to random gene expression and activation of the positive feedback loop in a sub-population of cells) when cells are exposed to intermediate concentrations of inducer. However, if those cells were previously exposed to high concentrations of inducer, a large fraction of cells will be in the ON state when transitioning to the new environment (with intermediate concentration of inducer), and the fraction of ON cells in this new environment may stay high for some period of time. In contrast, if those cells were previously grown in a medium without any inducer, (almost) all cells will be in the OFF state, and it will take some time before the first cells start to turn ON when exposed to intermediate concentrations of the inducer. Therefore, in these conditions, the probability of individual cells (hence the composition of the population) to be in the ON or OFF states depends not only on the current environment, but also on the history of environmental exposure. This effect was beautifully described in Ozbudak Nature 2004, among others. These behaviours can be interpreted as memory of the previous environmental conditions, encoded at the population level.
Yet, fitting into the definition of memory *sensu* Norman et al requires an additional level of complexity, stemming from the quantitative description of switching dynamics at the single cell level. Classical models of phenotypic switching consider that switching is a stochastic process occuring at a constant rate, which produces exponentially distributed waiting times. In these models, the probability to switch depends only on the current phenotypic state of the cell. However, the waiting time distributions of switching events have seldom been measured experimentally at the single cell level, preventing direct confrontation of switching dynamics with theoretical models. Using novel microfluidic setups, Norman et al. (Nature 2013) managed to image cells (grown under constant conditions) switching between 2 phenotypic states at sufficiently high throughput to describe waiting times distributions for both switching events, as well as the correlations between subsequent switching events. In their system, waiting times distributions were

asymmetric, following an exponential distribution in one direction, but a gamma distribution in the other. Gamma distribution of waiting times cannot be explained when switching occurs at a constant rate. Instead, switching rate must change depending on the time that each cell has already spent in the current phenotypic state. Therefore, they define memory as the fact that individual cells 'remember'/monitor the time that they already spent in a given phenotypic state to adjust cellular decision making and resulting in stereotypic waiting time. Switching behaviours occurring at a constant rate in a given phenotypic state are called 'memoryless'.

We have modified the introduction substantially so that we cover these points in providing a fuller justification for our use of the term memory. We hope this clarifies the issue.

5) This is up to the authors, but I found Figure S2 particularly clarifying: why not moving it to the main text and discussing it in more detail there?

Again, the reviewer has been perceptive here. The figure was indeed in the main text in a previous draft of our manuscript. We thought it might be an overload for the readers to get into the details right in the main text. Following the reviewer's advice though, we have moved the figure back in the main text and written it in so as to minimise the possible overload before moving to the Discussion. We hope the flow of the manuscript remains clear.

We thank the reviewer again for a careful reading of the manuscript and for the queries and suggestion which in our option have improved in clarifying the concepts presented in the manuscript.

Reviewer #2: In Gokhale et al, the authors describe an interesting and simple mathematical model of population-level phenotypic heterogeneity. In their model, cells can be in noone of two states: a default OFF state; and when (stochastically) triggered, the cells switch to an ON state. The key ingredient in the authors' model is the asymmetry in switching from OFF-to-ON versus ON-to-OFF. Specifically, the latter transition occurs slowly, as each cell transitions through a fixed number, n, of intermediate (but still ON) states, each at a leaching rate, epsilon, before they turn OFF. The authors claim that by tweaking n and epsilon, the two main parameters of the model, they can explain various phenomena observed in natural populations, such as the presence of antibiotic persisters, oscillations in the fraction of ON cells, and wide lag time distributions.

I certainly appreciate the model and I also agree with the authors that the role of memory in affecting microbial populations remains relatively under-explored. I think it will ultimately be a valuable contribution to the field. However, I have several concerns about the authors' claims and results that I think need to be addressed before this paper is published.

We thank the reviewer for seeing the value in our work and hope that what follows as well as the changes made in the manuscript addresses the queries.

Major concerns

1. Importance of multiple compartments: The authors claim that their model extends previous work by introducing several intermediate states (compartments) that cells must pass through while transitioning from ON to OFF, in this way going beyond models with just two binary states. While I understand this, I am still uncertain to what extent the authors need a model with multiple compartments to explain the key takeaways of the paper. In my understanding, even with just two compartments, the authors could have directly controlled the waiting time distribution, both its mean and width, and obtained very similar results. Moreover, it would still take the same number of parameters: two (one each for a mean and width) to describe such a model. I kept wondering why a multi-compartment model was necessary while reading the paper. I could argue about most results intuitively (the presence of persisters, for instance), and even the most interesting result (the phase diagram in Fig. 3) was something that seemed to work with a two compartment model in my head.
The authors claim that "adding compartments intensifies transients", but as I will discuss in a later point, this part of the paper needs some work, especially (for me) being better motivated biologically.

I therefore invite the authors to more carefully and explicitly explain which aspects of their results require multiple compartments, and which aspects can suffice with just two compartments, through direct control of the waiting time distribution in switching from the ON to OFF states.

If we understand correctly, Reviewer 2 suggests that multiple compartments could be redundant, as a 2 compartment model where mean and variance in the time spent in the compartment are specifiable parameters may just do the job. We admit that, in the

beginning, we were thinking precisely in those terms: 2 compartments with modified waiting times would suffice. However, we soon realized that in order to get such a model we would need to give up linearity. In fact, when there are only two compartments and dynamics are linear so that there is a constant flow between compartments, then the time a cell takes to move from one compartment to the other is exponentially distributed with mean, say 1/lambda, that cannot be chosen independently from the standard deviation, 1/lambda, for an exponential distribution. In order to have two compartments only while also modulating the switching rate depending on the time already spent in a compartment requires non-linear terms, e.g. in the form of delay differential equations, but there may be countless possibilities to hardwire a timekeeping mechanism via non-linearities. An important aspect of our work is that the introduction of multiple compartments underlying the 2 states preserves linearity: we can represent our model as a system of linear differential equations in matrix form where the matrix is constant. However, the time it takes to go from one state to the other in our model is no longer exponentially distributed. In fact, the time it takes to go from any one ON compartment to the OFF compartment is gamma distributed due to a sum of exponentials. We see this as a virtue of our model, as linearity is often a synonym of simplicity in modeling as appreciated by the reviewer right at the beginning. We now underline this aspect in the resubmission (In the discussion). Moreover, gamma distributions appear in experimental settings where similar systems are studied (but see more on this below). It would also seem that multiple compartments are easier to envisage to have a biological counterpart as opposed to a complicated nonlinear system of two differential equations.
[As a side note, we would like to first note that Reviewer 1 prompted us to consider a recently published paper in PLOS CB (Himeoka and Mitarai 2021) that also considers multiple compartments for two-state cell populations. However, we believe that the use we make of multiple compartments is very different from that of Himeoka and Mitarai 2021 (see our reply to Reviewer 1).]
We agree with the reviewer that in principle we could just shift the distribution of the time spent in an appropriate manner and recover different results. While we first thought of that approach, it leads to the next question - why is the distribution different? Where do the values of the changed parameters come from? Hence we decided to adopt a mechanistic point of view. For example in the new Himeoka and Mitarai paper similar question could be asked... where does λ come from? Furthermore, while the equilibrium behaviours would be replicated using the adjusted waiting times and hence the transition probabilities, the transients are not explained if we compress the information into two states assuming deterministic dynamics. This connects the next section on why we

motivate our work on a mechanistic basis rather than assuming ad hoc differences in waiting times. If we hardwire a waiting time distribution then it will be possible to derive complex dynamics in the transients depending on the choice of the distribution. However, this does not reflect why a particular choice is made. We do not make such an assumption but rather focus on a mechanistic process such as a degrading protein which we hope is a measurable variable that can lead us to a waiting time distribution. As for a biological justification, inspired by the observations and the analysis by Norman, T. M., Lord, N. D., Paulsson, J. & Losick, R. Memory and modularity in cell-fate decision making. Nature 503, 481–486 (2013) we assume that the cells need to complete an internal "to do" list before they can switch. Experimentally this could be compared to the concentration of a protein to be eluted out of the cell. Therefore thought our figures and analysis constantly compared our multicompartment results with a two-compartment case to highlight the differences.

2. Biological basis for microbial memory: The authors argue that phenotypic memory could be realized via distinct internal states, such as through the concentration of an internal molecule. This to me needed much more discussion and a lot of biological motivation throughout the text. While I find it easier to imagine memory being realized through multi-cell circuits in eukaryotes, I find it quite hard to think about them in unicellular populations. More direct examples, such as discussing specific studies and putting them in context would really help.

We agree that providing more biological background would strengthen our manuscript. A more detailed definition of memory has now been added to the introduction (see response to reviewer 1). Although highly desirable, more detailed examples on the correspondence between molecular mechanisms and switching behaviours are actually difficult to provide (but see below). This is because there are very few examples of biological systems were both the molecular mechanisms and the dynamics of switching behaviours were described with enough detail. The most convincing case can be made on the switch between motile and sessile states in Bacillus subtilis. Norman et al. (2013) measured the waiting times distributions in both states, and Lord et al. (2019) identified the critical features of the regulatory network that are responsible for memory. In short, the sessile state (showing memory) occurs when the protein SinR (a repressor of sessile-associated genes) is titrated by the protein SinI. Stochastic burst of SinI production leads to memoryless switching to the sessile state (where [SinI] > [SinR]). A combination of two phenomena generates memory and stereotypical waiting times before switching back to

motile state (where [SinI] < [SinR]). Dilution at cell division leads to a gradual decrease in SinI concentration. When SinI concentration falls below the level of SinR (produced at a constant rate), free SinR will inhibit the expression of sessile-associated genes, leading to the switch to the motility state. Thus, the titration of the remaining SinI proteins prevents stochastic fluctuations that would have been associated with the dilution of rare molecules. Dilution at cell division ensures that the variability of SinI dominance periods is small, since when a burst leads to the production of twice as many SinI proteins, this difference only takes 1 additional cell division to be diluted. This is now included in the Introduction, lines 43-46.
Another type of example are the gamma distributions of waiting times during lag phases, that were described in a few papers (Moreno Gamez PNAS, Fridman Nature 2014). However, the exact molecular mechanisms are not completely clear (and likely differ between the different types of lag phases). In theory, any cases where a combination of stochastic events with similar time scales (otherwise, the dynamics of the slower process will dominate; see Norman 2015) should produce gamma distributions of residence times. For example, multisite protein modifications can generate stereotyped waiting times before triggering a response ((cite Salazar et al 2010 https://doi.org/10.1016/j.tcb.2010.08.012; Upadhyay et al 2020 https://doi.org/10.1038/s41598-020-79277-z; Bel et al 2009 https://doi.org/10.1088/1478-3975/7/1/016003; Doan et al 2006 10.1126/science.1126612

Finally, excitable switches may also generate gamma distributions of waiting times, but, to our knowledge, accurate switching dynamics in such systems have not been published.

We thank the reviewer for making us think more about this aspect and we modified our manuscript particularly in the introduction to highlight these biological considerations as well as to better motivate our approach. Our hope is that the theoretical model that we propose in this manuscript may promote the designing of experiments and foster dialogue between experimental and computational biologists about elucidating the type of mechanism involved in phenotypic switching,

I therefore invite the authors to juxtapose the results of their model with real biological observations not just throughout the text, but also more explicitly, by discussing the relevant numbers and representing the observed phenomena in the figures. This could be

done, for instance, by showing real lag time distributions and comparing their features with those produced by the model.

This comment by the Reviewer was important for us in reconsidering the ability of our model to replicate experimental results directly. We are persuaded that it is important that our model can produce gamma-distributed lag times, like in e.g. Moreno-Gamez et al PNAS 2020, in certain conditions, i.e. when cells are sampled from one of the ON compartments. . Notably, in these experiments cells are first let grow with abundant media, then they are let reach a stationary phase to be then sampled from there to resume growth in abundant media. Growth dynamics (exponential-stationary-exponential) in this setting are not replicated in our model, where death and birth rates are minimized on purpose to focus on the dynamics of phenotypic switching.
As we cannot envisage an obvious way of mapping the experimental setting with the model, we have toned down the relevant sections in the new submission and specified that a gap exists between our work and the experiments which can only be filled when we decide to focus on a particular model system.

Further, the authors should give some concrete examples of realizing memory and relating it with microbial phenotypes. How should one think of multiple discrete phenotypic states in the case of antibiotic persisters? Which molecules should we think of as examples? I understand that the model is quite general, and as such does not describe a particular molecule or cell, but it would be nice to ground it in the context of a few real and biologically motivated examples.

Given the changed introduction as well as the discussion we hope that the first part of the comment on realising memory has been addressed. As for the role of multiple compartments, we take inspiration for the “to-do” list approach of Norma et al. While certain studies are moving in the direction of multiple compartment (Himeoka and Mitarai) they are theoretical or computational. Apart from the Norman study, an emerging area where multiple phenotypic states are extremely important to understand is cancer. Understanding the mechanisms allowing transitions between these states might be crucial in designing therapeutic strategies (Jia, D. *et al.* Elucidating cancer metabolic plasticity by coupling gene regulation with metabolic pathways. *Proc National Acad Sci* **116**, 201816391 (2019).). Connecting such studies to the presence of memory as we use it, is possible (Sellerio, A. L. *et al.* Overshoot during phenotypic switching of cancer cell populations. *Sci Rep-uk* **5**, 15464 (2015).). While we see that the number of studies focusing on this particular aspect are sparse, our hope is that our work will spark an

interest in viewing the switching process as a continuum of multiple discrete states. We refer to these studies in the discussion now to ground our results.
Along with our updated introduction and discussion where we focus more on specific examples, we hope that we have delved deep enough into the biological motivation and connections to observations for our computational study.

3. Transient damped oscillations: The section titled “Transient properties”, to me, represents the biggest opportunity for the authors. Here, they can talk about the damped oscillations and show what they look like in real systems. In the Discussion, the authors talk about circadian clocks and other oscillations, but in my mind they are quite different from such damped oscillations. I think the mechanism of how the overshoots and undershoots occur should also be explained somewhat more through cartoons in Fig. 2, rather than just showing the phenomenon. Moreover, this figure generally needs work, because I could not find the relevant legends anywhere, so I didn’t know what the colors in Fig. 2A mean.

Indeed, the Transient properties section is where the effect of the compartments is highlighted and we appreciate that the reviewer sees the importance of this section. The damped oscillations here are for the ON-OFF cells and the time for relaxation to equilibrium is connected to the eigenvalue analysis presented in the SI.
The external cycles and oscillations as mentioned in the Discussion however do not connect to the oscillations we see in the ON-OFF cells. They are meant to capture natural oscillatory environmental conditions in comparison to the antibiotic treatment schedules as we described in our toy example. Hopefully we have better explained this in the manuscript now.

Additionally, in general, since the authors claim that multiple compartments are crucial for such transients to occur, I think this section needs to do a lot more to convince the reader that this really is a phenomenon we need to explain, and that two compartment models of various kinds are insufficient. For instance Fig. S1 shows that with n=1 compartment, one does not obtain transients, but that’s when there is a fixed leaching rate, epsilon. How about the two compartment model I described, where the mean and variance of the waiting time distribution can be tuned independently? Would that be able to obtain transients? I am sorry for belaboring this point, but I would really like to know, since I think the authors have something interesting here.

We agree with the Reviewer that a comparison between 2 compartments and > 2 compartments is crucial to our work. However, we would like to underline that all our figures are meant to highlight precisely this. In virtually all figures, this is done by looking at the case n=1 vs n>1. Whenever possible, we make the comparison in their performance in the transient phase as fair as possible by making the two models asymptotically equivalent.
As for comparing the present model with the approach recommended by the reviewer we again refer to our reply above. Already including a different waiting time distribution includes the solution in the assumption itself, it involves a more complicated mathematical machinery and does not provide a mechanistic argument.

Minor concerns
1. What do the curves in Fig. S7 represent? Are they trajectories? If so, where do they start? I had a hard time understanding this figure, but I also think it might be potentially interesting. The reason is that it is still unclear to me whether, for memory to be beneficial, the antibiotic episode needs to be one long sustained episode, or whether it is the total amount of time the antibiotic was present that matters. Fig. 3 alone cannot answer this, but Fig. S7 might. Naively, I would think that the duration of an episode is important, since that is what you need to tune, but if you have multiple intermittent episodes very often, then it might be useful as well.

We apologise for the inadequate caption of Fig. S7 -> now S6. We have now expanded on the caption. While indeed the duration of the consecutive treatment is important, our figure shows that it is also important as to when that occurs. For example, comparing the top left and the bottom right permutation - the treatment happens consecutively for four seasons. However, in the top left the sequence starts with the treatment while in the bottom right it ends with it. Considering the final frequency of on cells, more memory is then better irrespective of these two scenarios but the final population size is larger for larger memory when treatment is immediate whereas shorter memory is better if treatment is late.

We thank both the reviewers for the excellent and though-provoking queries and we do feel the changes we have made in the manuscript do indeed help make our study stronger and more grounded at the same time. We hope you concur.

Dynamics of the model with differentiation

Include cell differentiation where reproduction happens in the next compartment

The model tracks the movement of the cells from compartments $X_n \rightarrow X_{n-1} \rightarrow X_{n-2} \rightarrow \ldots \rightarrow X_0$
All cells in compartment X_0 are *off* cells which all others are *on*
The switch from $X_0 \rightarrow X_n$ occurs with probability μ

In this notebook we extend the model to include differentiation. Thus each cell can thus undergo four processes:

1. Differentiation
   $x_i \rightarrow x_{i-1} + x_{i-1}$ with probability ϵ
2. Death
   $x_i \rightarrow \phi$ with probability d
3. Switch to X_n
   $x_i \rightarrow x_n$ with probability μ (valid only for $i = 0$ and $\mu = 0$ otherwise)
4. Reproduce
   $x_i \rightarrow x_i + x_i$ with probability (1-ϵ-d-μ)

As compared to the main text now we represent the rates with probabilities of each process to materialise.
Thus the interpretation of the rates changes but the essence of the model stays the same.
The deterministic dynamics of the process can then be described by the following three equations:

$\dot{x}_n = x_n(1 - 2\,\epsilon - 2\,d) + \mu\,x_0$
$\dot{x}_i = x_i\,(1 - 2\,\epsilon - 2\,d) + 2\,\epsilon\,x_{i+1}$ (for $i = 1,\ldots,n-1$)
$\dot{x}_0 = x_0(1 - 2\,d - 2\,\mu) + 2\,\epsilon\,x_{n-1}$

Running the model for three cycles, each lasting for 100 time-steps.

The plots below show the population size in the on and off compartments for different leaching rates (rows) and different memory sizes (5,15,25,35). This corresponds to the main text figure *Transient dynamics in multi-state memory (b).*

Out[81]=

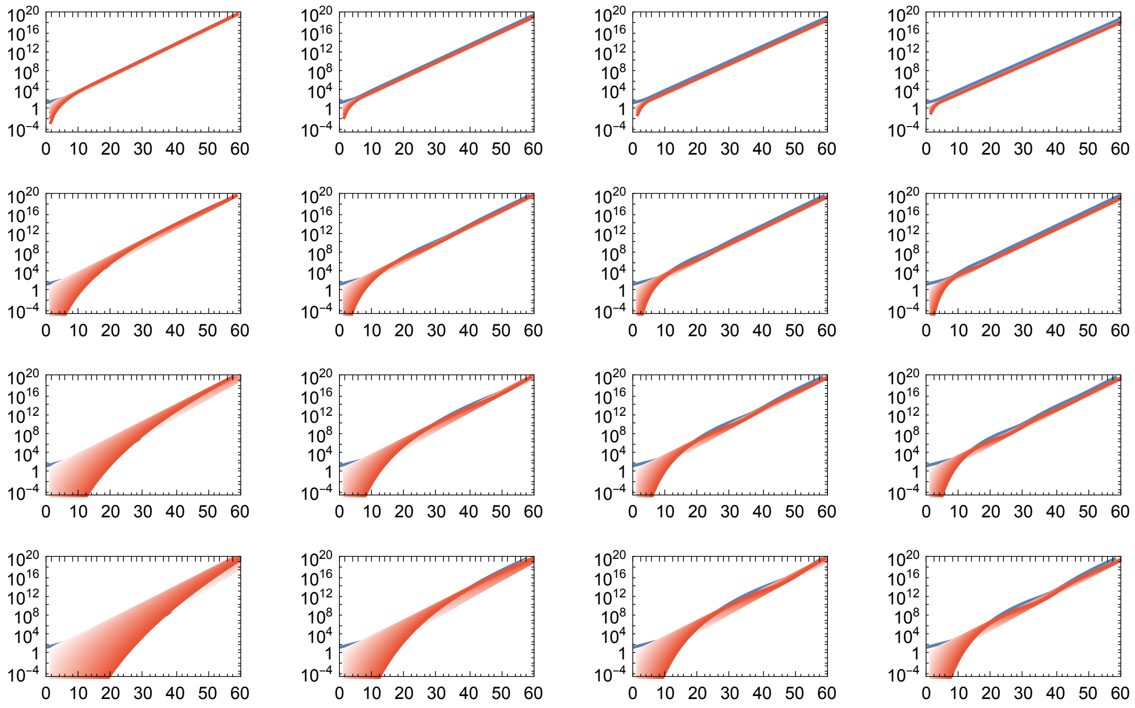

Transient dynamics for different number of compartments

The plots show the increase in over(under) shoots as the number of compartments is increased from 5, 15, 25, 35

Out[44]=

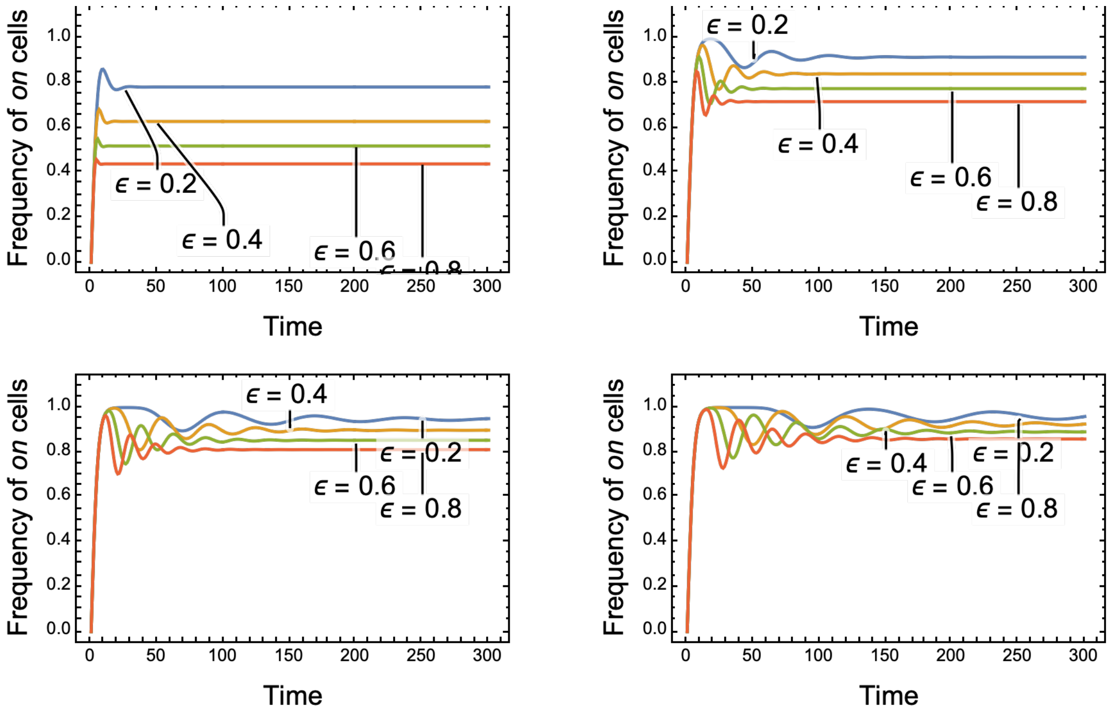

Transient dynamics of multi-state memory for a given ϵ

Focus on only the first cycle which runs for 100 time steps.

Out[45]=

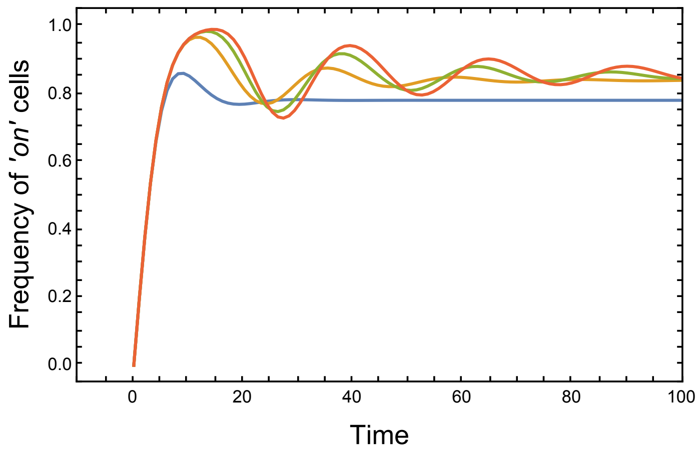

Supplement: Attachment — Submitted filename: replytoreviewers.pdf [file pcbi.1009431.s002.pdf]
